# Enhanced Glutamatergic and Decreased Gabaergic Synaptic Appositions to GnRH Neurons on Proestrus in the Rat: Modulatory Effect of Aging

**DOI:** 10.1371/journal.pone.0010172

**Published:** 2010-04-14

**Authors:** Mohammad Khan, Liesl De Sevilla, Virendra B. Mahesh, Darrell W. Brann

**Affiliations:** Institute of Molecular Medicine and Genetics, Developmental Neurobiology Program, Medical College of Georgia, Augusta, Georgia, United States of America; University of Córdoba, Spain

## Abstract

**Background:**

Previous work by our lab and others has implicated glutamate as a major excitatory signal to gonadotropin hormone releasing hormone (GnRH) neurons, with gamma amino butyric acid (GABA) serving as a potential major inhibitory signal. However, it is unknown whether GABAergic and/or glutamatergic synaptic appositions to GnRH neurons changes on the day of the proestrous LH surge or is affected by aging.

**Methodology/Principal Findings:**

To examine this question, synaptic terminal appositions on GnRH neurons for VGAT (vesicular GABA transporter) and VGLUT2 (vesicular glutamate transporter-2), markers of GABAergic and glutamatergic synaptic terminals, respectively, was examined by immunohistochemistry and confocal microscopic analysis in young and middle-aged diestrous and proestrous rats. The results show that in young proestrous rats at the time of LH surge, we observed reciprocal changes in the VGAT and VGLUT2 positive terminals apposing GnRH neurons, where VGAT terminal appositions were decreased and VGLUT2 terminal appositions were significantly increased, as compared to young diestrus control animals. Interestingly, in middle-aged cycling animals this divergent modulation of VGAT and VGLUT2 terminal apposition was greatly impaired, as no significant differences were observed between VGAT and VGLUT2 terminals apposing GnRH neurons at proestrous. However, the density of VGAT and VGLUT2 terminals apposing GnRH neurons were *both* significantly *increased* in the middle-aged animals.

**Conclusions/Significance:**

In conclusion, there is an increase in glutamatergic and decrease in GABAergic synaptic terminal appositions on GnRH neurons on proestrus in young animals, which may serve to facilitate activation of GnRH neurons. In contrast, middle-aged diestrous and proestrous animals show a significant increase in *both* VGAT and VGLUT synaptic terminal appositions on GnRH neurons as compared to young animals, and the cycle-related change in these appositions between diestrus and proestrus that is observed in young animals is lost.

## Introduction

The reproductive system is one of the first systems in the body to show age-related dysfunction in females which leads to an eventual loss of cyclicity and fertility. Prior to becoming acyclic, normal cycling middle-aged rats display a significantly *attenuated* proestrous GnRH and LH surge, which is thought to contribute to the age-related decline in reproductive function [1], [2], [3], [4]. The number of GnRH neurons is not changed in middle-aged rats, and the secretory apparatus appears intact, as naloxone and KCl-stimulated GnRH release from middle-age hypothalami is similar to that observed from young hypothalami [5], [6], [7], [8]. However, activation of GnRH neurons on proestrus in the middle-aged rat is significantly *attenuated* as evidenced by a significant reduction in c-fos and c-jun expression in GnRH neurons in middle-aged versus young proestrous rats [9], [10], [11]. Le et al. [12] further confirmed that there was an approximately 50% decrease in the number of GnRH neurons expressing c-fos in middle-aged rats as compared to young rats during the proestrous and steroid-induced LH surge. The results of these studies have been interpreted to suggest that GnRH neurons are not being activated as highly in middle-aged proestrous rats during the LH surge as compared to young proestrous rats, potentially explaining the significantly reduced LH surge in middle-aged rats.

However, it remains unclear as to why GnRH neurons are not being activated properly on proestrus in middle-aged rats. Possible explanations could include altered synaptic control of GnRH neurons that leads to their inhibition or diminished response to excitatory signals. Two major transmitter systems that control GnRH secretion are the excitatory transmitter glutamate and the inhibitory transmitter gamma amino butyric acid (GABA) [13], [14], [15], [16], [17]. Agonist, antagonist, and anatomical studies by our laboratory and others have implicated glutamate as a principal excitatory signal that stimulates GnRH secretion and GABA as a principal inhibitory signal to restrain GnRH release [13], [14], [15], [16], [17]. Intriguingly, our laboratory has demonstrated that middle-aged female rats exhibit a significantly decreased GnRH neurosecretory response to glutamate agonists on proestrous afternoon as compared to young proestrous rats [4]. There is also evidence that GABA production in the POA decreases on the afternoon of proestrus during the LH surge in young rats, an effect that is lost in middle-aged rats [18]. In addition, work by Han et al [19] using gramicidin perforated-patch electrophysiology in GnRH-LacZ and GnRH-GFP transgenic mice found that the majority of GnRH neurons examined responded to the selective GABA-A receptor antagonist, bicuculline with a rapid and reversible membrane depolarization and/or increased firing rate, suggesting that endogenous GABA inhibits firing of adult GnRH neurons. However, in contrast to the above studies, several groups have provided evidence that GABA may have an *excitatory* effect upon GnRH neurons, as rapid activation of GABA-A receptors was shown to excite GnRH neurons in GnRH-GFP transgenic mice and rats [20], [21] and in immortalized GnRH neurons [22], [23]. A *biphasic* effect of GABA on GnRH neuron excitability has also been reported in which there is an initial rapid enhancement of firing, followed by a prolonged suppression [20], [23], [24]. In addition, GABA-B receptor activation has been reported to hyperpolarize GnRH neurons GnRH-GFP transgenic mice [25], suggesting further complexity in GABAergic regulation of GnRH neurons.

Recent studies have identified dual phenotype GABAergic/glutamatergic cells in the anteroventral periventricular nucleus (AVPV) that make appositions to GnRH neurons [26], [27], [28]. Additional studies demonstrated that during the estradiol-induced LH surge, there is a decrease in GABAergic synaptic terminals (as determined by staining with vesicular GABA transporter, VGAT) contacting GnRH neurons, while there was an increase in contact by glutamatergic terminals (as determined by staining with vesicular glutamate transporter-2, VGLUT2) [28]. However, to date, no studies have been performed to assess GABAergic or glutamatergic contacts on GnRH neurons in natural cycling animals during the preovulatory LH surge. Additionally, it is unclear as to why there is an age-related decline in GnRH activation on proestrus in middle-aged rats. We hypothesized that alteration in GABAergic and glutamatergic contacts on GnRH neurons in middle-aged animals may underlie the decreased GnRH neuronal activation and decreased LH surge observed in middle-aged rats. Thus, the studies described herein were designed to address whether alterations in GABAergic and glutamatergic contacts on GnRH neurons occurs during the estrous cycle and with aging in female rats.

## Materials and Methods

### Animals

The animals used in this study were Sprague Dawley rats were obtained from Harlan, IN. Animals were maintained according to NIH Guidelines for the Care and Use of Laboratory Animals, and the Institutional Animal Care and Use Committee of the University of Georgia approved all treatment protocols. Animals were housed in a temperature- and light-controlled room (14/10 hr light/dark cycle; lights on at 6:00 A.M.) with food and water provided *ad libitum*. Two-three months old young naïve female rats and 9–11 month old retired breeder middle-aged female rats were used in the study. The number of animals used per groups is as follows: young diestrus (n = 6), young proestrus (n = 7), middle-aged diestrus (n = 5) and middle-aged preostrus (n = 6). These results were repeated twice using same number of animals. We also used 3–4 month old, one time breeder as young proestrus (n = 6). Regular 4-d cycling animals were killed on proestrous or diestrus (1500–1700 h) and tissue processed for Western blot or immunohistochemistry. Only animals that showed at least two-three consecutive 4-d cycles were included in the study. Cycle stages were determined by daily recording of electrical impedance of the vaginal wall using a commercially available EC40 estrous cycle monitor (Fine Science Tools, Foster City, CA) [29]. Vaginal lavage and microscopic determination of vaginal cytology was also used to confirm estrus cycle stages.

### Western Blot Analysis

For Western blot analysis, tissue was homogenized in RIPA buffer (1X Phosphate Buffered Saline, PBS), 1% IGEPAL CA-630 (Sigma Chemicals, St. Louis, MO), 0.5% sodium deoxycholate, 0.1% SDS) and protease inhibitors using a polytron homogenizer. After homogenization, samples were centrifuged at 10,000 rpm for 10 min at 4°C. The supernatant fraction was the total cell lysate. Protein concentration in the lysate was determined using a total protein measurement kit from Sigma Chemicals. Protein samples were denatured in sample buffer containing β-mercaptoethanol in 25 mM Tris-glycine buffer and separated on a sodium dodecyl sulfate-polyacrylamide gel after loading equal amount of protein in each lane. Separated proteins were transferred to Immobilon P membrane (Millipore, Billerica, MA, USA) at 27 V for 15 h in 25 mM Tris-glycine buffer, pH 8.3, 10% methanol using a Transblot apparatus (Bio-Rad Laboratories, Inc., Hercules, CA). Following the transfer, the membranes were rinsed twice with T-TBS (20 mM Tris, 137 mM NaCl, 0.1% Tween-20) for 5 min each rinse and then incubated with 5% non-fat dry milk for 1 h at room temperature to block non specific/unbound surface. The membrane was incubated overnight with either polyclonal guinea pig anti-VGAT (1∶2000; EMD Biosciences, Gibbstown, NJ) or monoclonal mouse anti-VGLUT2 (1∶1000; Millipore, MA). The membrane was then washed with T-TBS to remove unbound antibody, followed by incubation with secondary HRP-conjugated donkey anti-rabbit IgG (Transduction Laboratories, San Diego, CA) for 1 h at room temperature. The signal was detected using an ECL detection kit (GE Healthcare, UK) and the membranes were exposed to Kodak Biomax MR film.

### Immunohistochemistry for light microscopy

Tissue fixation: Animals were deeply anesthetized with 120 mg/kg ketamine/10 mg/kg xylazine and transcardially perfused with saline followed by fixation with ice-cold solution of 4% paraformaldehyde and 0.05% glutaraldehyde in 0.1 M phosphate buffer, pH 7.4. After perfusion, rodents brains were removed and placed in the same fixative overnight at 4°C, followed by incubation in 30% sucrose solution in 0.1 M phosphate buffer, pH 7.4 at 4°C until the brains sank. Tissue was frozen in OCT (optimum cutting temperature) compound under an atmosphere of nitrogen, and serial coronal sections (40-µm thickness) were obtained on a cryostat (Leica, Germany) through the POA region that contains the AVPV, the medial preoptic nucleus (MPO) and the periventricular POA (PePO) (−0.00 to −0.26 from bregma). The sections were stored in a cryoprotection solution (FD Neurotechnology, Inc, Baltimore, MD) in stereological order for immunostaining.

#### Silver-enhanced nanogold labeling

Free floating sections were rinsed in PBS containing 0.1% Triton X-100, and incubated with blocking solution containing 0.1% cold-water fish gelatin, 0.5% bovine serum albumin, and 5% normal donkey serum for 2 h at room temperature in the same buffer. Sections then incubated for 24–36 h at 4°C with the following primary antibodies; guinea pig anti-VGAT (1∶4000; Calbiochem, CA) and mouse anti-VGLUT2 (1∶3000; Millipore, MA). Afterward, antibody solution was removed by rinsing repeatedly with PBS containing 0.1% Triton X-100. For silver-gold staining, sections were then incubated with appropriate gold labeled donkey anti-rabbit or anti-mouse secondary antibody (ultra-small; Aurion, Electron Microscopy Sciences, Hatfield, PA) at a dilution of 1∶100 in PBS containing 0.1% Triton X-100 for 3 h at room temperature. After rinsing with PBS containing 0.1% Triton X-100 and with PBS alone, the sections were post-fixed in 2% glutaraldehyde diluted in PBS for 10 min followed by washing with PBS and distilled water. The sections were then processed with the prepared silver enhancement reagent (Aurion R-Gent Developer and Enhancer, Electron Microscopy Sciences, PA) at room temperature for 15–30 min. After rinsing with PBS and distilled water, the sections were mounted on slides, and dried. The sections were finally dehydrated in a graded series of ethanol, cleared with xylene, and mounted with xylene-based mounting medium (VWR Scientific, Atlanta, GA).

#### Image acquisition and analysis of silver-gold nanoparticles

Images from silver-enhanced nanogold labeled sections were captured on an Axiophot-2 visible/fluorescence microscope with 100X optical lens using AxioVision4Ac software system (Carl Zeiss, Germany). Approximately 10–12 sampling sites were obtained for each animal and for quantitation images were background subtracted, flat field divided, and interactively thresholded using a classifier to define various parameter of the gold particles. A single threshold chosen manually for all the images and the intensity of gold particles was analyzed using *Volocity4* (Perkin Elmer, Waltham, PA). The results were presented as mean density of gold particles per unit area (100 µm^2^) and statistical analysis was performed by one-way ANOVA with Bonferroni's posttest to obtained significance of variation between the groups.

### Immunohistochemistry for confocal microscopy

#### Immunofluorescence staining

Immunofluorescence staining was performed as described previously by our laboratory [30], [31]. Free-floating sections were incubated with 12% normal donkey serum for 2 h at room temperature in PBS containing 0.1% Triton X-100, followed by incubation with appropriate dilutions of the primary antibodies for 24–36 hr at 4°C in the same buffer. The primary antibodies used were monoclonal mouse anti-VGLUT2 (1∶3000; Millipore), polyclonal guinea pig anti-VGAT (1∶4000; Calbiochem) and rabbit polyclonal anti-GnRH (1∶2000; Millipore). The dilutions and concentrations of antibodies were selected based on previously studies in rodent brain documenting their specificity and selectivity [28]. After primary antibody incubation, sections were washed for 4×10 min at room temperature followed by incubation with Alexa-Fluor488 donkey anti-mouse, Alexa Fluor647 donkey anti-rabbit and TRITC-labeled donkey anti-guinea pig IgG antibodies (1∶500) (Molecular Probes, Invitrogen Corporation, Carlsbad, CA) for 1 h at room temperature. Sections were then washed with PBS containing 0.1% Triton X-100 for 4×10 min, followed by 3×5 min with PBS and 2×1 min with water, and then mounted with water-based mounting medium containing anti-fading agents (Biomeda, Fischer Scientific, Pittsburgh, PA). A simultaneous examination of negative controls (omission or blocking of primary antibody with antigenic peptide confirmed the absence of nonspecific immunofluorescent staining, cross-immunostaining, or fluorescence bleed-through.

#### Image acquisition and analysis of VGAT and VGLUT2 synaptic terminals

All the double and triple-labeled images were captured on an LSM510 Meta confocal laser microscope (Carl Zeiss, Germany) in XYZ plane using a 60X oil immersion Neofluor objective (NA, 1.3) with additional 3X digital zoom and the image size was set at 512×512 pixels. The Z-stacks (20–25 optical slices) were obtained at a thickness of 0.5 µm using optimum pinhole diameter with 12-bit pixel depth. The Z-stacks were then converted into 3D projection or Stereo image using LSM 5 Image Examiner. For excitation/emission of various chromophores, we used the following excitation lasers and emission filters: Argon/2 (for Alexa Fluor488, Ex: 488, Em: 505–530), HeNe1 (for TRITC, Ex: 543, Em: 568–615) and HeNe2 (for Alexa Fluor647, Ex: 633, Em: 650–800). For the counting of VGAT and VGLUT2 synaptic terminals apposing GnRH neurons, we used Zeiss LSM 5 Image Browser software that allows virtual rotation of cells to verify that terminals actually contact GnRH neurons in multiple fields of view. Counting of terminals was also performed using Volocity 4 software or manually after tracing each GnRH neuron and then converting 3D projection images into JPEG files.

For each animal, 10–12 cryostat microtome sections (40 µm thick) were collected encompassing the entire rostral preoptic area and were arranged in chronological order. From these 10–12 cryostat sections, we analyzed 5 or 6 alternate sections starting from the 1^st^, and no more than two GnRH neurons per section were carefully traced and scanned using confocal microscopy as discussed above. In this way we obtained 10 (5 sections×2 neurons) or 12 (6 sections×2 neurons) GnRH neurons per animal. All the GnRH neurons were selected only from the medial part of the rostral preoptic area within the well delineated stereotaxic coordinates, so in all animal groups GnRH neurons were selected from the same region of the rostral preoptic area. Finally, we took the average of all 10–12 neurons for each animal used in the statistical analysis; therefore, each animal represents a unit of analysis. For statistical analysis, mean values of VGAT and VGLUT2 terminals were obtained for each animal and analyzed by either two-way ANOVA or one-way ANOVA using GraphPad Prism (version 5.03). To obtained significance of variation and interaction between the groups, if any, we applied either Bonferroni's posttest analysis or Student Newman-Keuls posttest analysis depending on the number of variables. A p<0.05 was considered to be significant. All the experiments were repeated twice keeping the number of animals more or less same and the results were identical in both the experiments.

## Results

### Expression of VGAT and VGLUT2 transporter proteins in the rPOA

**Figure 1:**
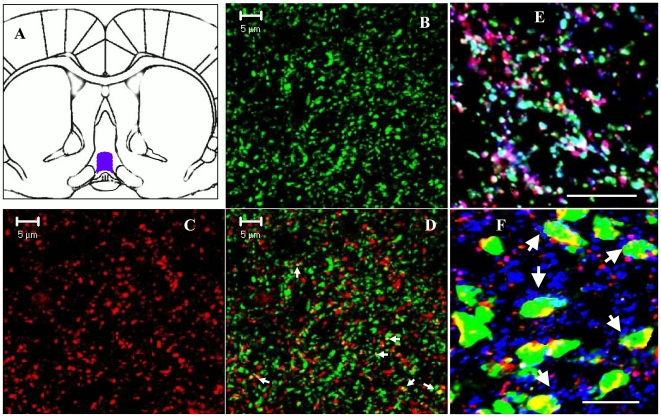
Expression of VGAT and VGLUT2 synaptic proteins in the rPOA of the proestrous female rat. Figure 1A shows a schematic diagram of rat brain in coronal plane highlighting the POA region. Figures 1 B and C show 3D confocal images of VGLUT2 (green) and VGAT (red) obtained using 63X objective lens with additional 3X digital zoom. Both VGLUT2 and VGAT show punctate staining characteristics of presynaptic terminals. Figure 1D shows a merged (colocalized) image which indicates that while both VGLUT2 and VGAT proteins are highly expressed in the same region, only a small percentage (∼10%) of them show colocalization (white arrows) with each other. Figure 1E shows an enlarged view of VGAT (red) and VGLUT 2 (green) colocalization (>90%) with synaptophysin (blue) in the POA region. Figure 1F shows that almost all ERα positive (green) neurons show colocalization/co-expression (white arrows) of VGAT (red), VGLUT2 (blue) in the AVPV region. Scale bars represent 5 µm in Figure 1 B-D and 10 µm in Figure 1 E-F.

Recent observations in ovariectomized rats suggested that inhibitory and excitatory projections from ERα-containing AVPV neurons terminate into the rPOA, which harbors GnRH neurons sensitive to E2 and photoperiodic signal [28]. Through these projections, AVPV neurons are proposed to provide GABAergic and glutamatergic synaptic transmission to GnRH neurons and regulate GnRH release during the estradiol-induced LH surge in ovariectomized animals [28]. To verify that the terminals of these inhibitory and excitatory projections express VGAT and VGLUT2 synaptic proteins in the normal intact cycling animal on *proestrus*, the day of the natural preovulatory LH surge, a double immunofluorescence labeling was performed on the coronal sections of proestrous animals passing through the rPOA. Several Z-series images were captured from the rPOA of proestrous female rats and converted into 3D projection images for analysis. **Figure 1A**, shows the region of rPOA which was first scanned at low resolution using 20X objective lens followed by scanning using 63X objective lens with additional 3X digital zoom. As shown in **Figure 1B & C** **,** both VGLUT2 and VGAT, respectively, are highly expressed in the rPOA, the region which harbors GnRH neurons critical for the LH surge. Furthermore, VGAT and VGLUT2 staining both show a punctuate staining pattern; a common feature of presynaptic proteins. Also, as shown in **Figure 1D** **,** both VGAT and VGLUT2 proteins are highly expressed in the same region, but only a small number (<10%) of them show colocalization (*white arrows*). This suggests that a majority of VGAT and VGLUT2 positive terminals provide GABAergic and glutamatergic transmission to the rPOA in an *autonomous* manner. It should be mentioned that the majority of VGAT and VGLUT2 positive terminals (>90%) showed absolute colocalization with the synaptic marker, synaptophysin (SYN) (Figure 1E), which agrees with previous work demonstrating synaptic localization of VGAT and VGLUT2 [32], [33]. Furthermore, an extensive analysis revealed an identical pattern of VGAT and VGLUT2 punctate staining in cycling young and middle-aged female animals at diestrus or proestrus, however; the density of both VGAT and VGLUT terminals was significantly altered in the rPOA of the middle-aged animals as discussed afterward. It is important to mention that VGAT and VGLUT2 projections, which terminate into rPOA are suggested to originate from ERα containing AVPV neurons which express markers of inhibitory (VGAT) as well as excitatory (VGLUT2) synaptic transmission (29). To verify that these ERα positive AVPV neurons actually express VGAT and VGLUT2 markers, we performed confocal microscopy on brain sections triple labeled for VGAT, VGLUT2 and ERα markers. The result presented in Figure 1F, show that virtually all ERα positive neurons (green) from AVPV region express both VGAT (red) and VGLUT2 (blue) proteins, which are in agreement with previous findings (29).

### Organizations of VGAT and VGLUT2 synaptic terminals onto GnRH neurons

**Figure 2:**
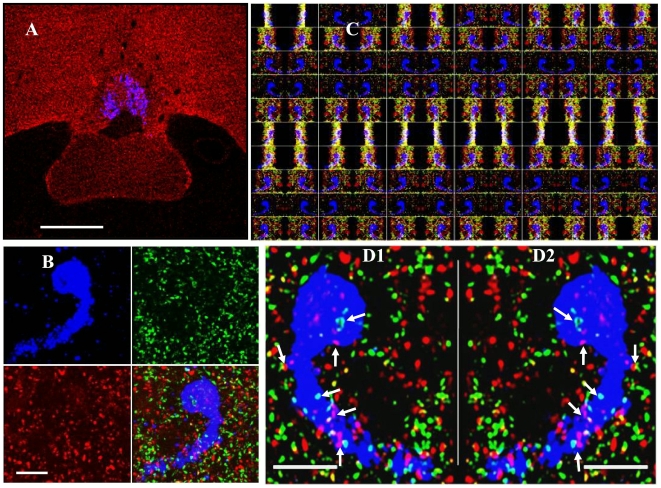
Organization of VGAT and VGLUT2 synaptic terminals onto GnRH neurons. Figure 2A, a low magnification confocal image of the brain section, encompassing POA regions, stained for VGAT (red), GnRH (blue) and VGLUT2 (green; channel hidden for clarity). Figure 2B is a *3D* confocal image created using Z-stacks. Figure 2C shows stereo views of a single GnRH neuron in various projections (60 projections). Figures 2 D1 and D2 show magnified stereo images of a GnRH neuron and organization of VGAT (red) and VGLUT2 (green) synaptic terminals. A synaptic terminal apposing GnRH neuron was confirmed and counted only when it apposed the cell body and dendrite (white arrows) throughout the rotation of the cell in multiple fields around Z axis. Scale bars represent 50 µm in Figure 2A, and 10 µm in Figure 2B and D.

The projections from AVPV neurons which terminate in the rPOA may form inhibitory (VGAT-positive) or excitatory (VGLUT2-positive) synaptic terminals. To get an insight into how these terminals are organized *three dimensionally* (*3D*) in rPOA and in apposition with GnRH neurons, we made a comprehensive analysis of the *3D* images captured using confocal laser microscopy from the sections triple-stained for VGAT, VGLUT2 and GnRH markers. **Figure 2A** shows a low magnification confocal image encompassing POA regions of the brain section stained for GnRH (blue), VGAT (red) and VGLUT2 (green; not shown for clarity). The box in **Figure 2A** shows the rPOA region which was scanned at higher magnification using three different excitation wavelengths and is depicted in **Figure 2B**. Several individual GnRH neurons were scanned from rPOA using Z-series scanning (20–25 Z-stacks) encompassing the whole cell body and dendrite and converted to 3D projection or stereo image as shown in the **Figure 2C** and **D** respectively. The stereo images shown in **Figure 2 D1** and **D2** are the result of virtual rotation at 180° around Z-axis in Z-Y-Z plane. As can be seen in **Figure 2 D1**, a large number of VGAT and VGLU2 positive synaptic terminals (*white arrows*) directly appose the GnRH cell body and the spiny protrusions from the dendrite. Furthermore, the *three dimensional* arrangement of VGAT and VGLUT2 terminals apposing GnRH was confirmed by virtual rotation of the cell in multiple fields of view as shown in **Figure 2 D2**. A synaptic terminal apposing GnRH neuron was confirmed and counted only when it juxtaposed the cell body and dendrite throughout the rotation of the cell in multiple fields. Additionally, as can be seen in **Figure 2D**, some of the VGAT- and VGLUT2-positive synaptic terminals superimpose (colocalize) with each other in and around the GnRH neurons in the rPOA, which were counted both as VGAT-positive and VGLUT2-positive terminals.

### Cycle changes in VGAT and VGLUT2 synaptic terminals apposing GnRH neurons and the effect of aging

**Figure 3:**
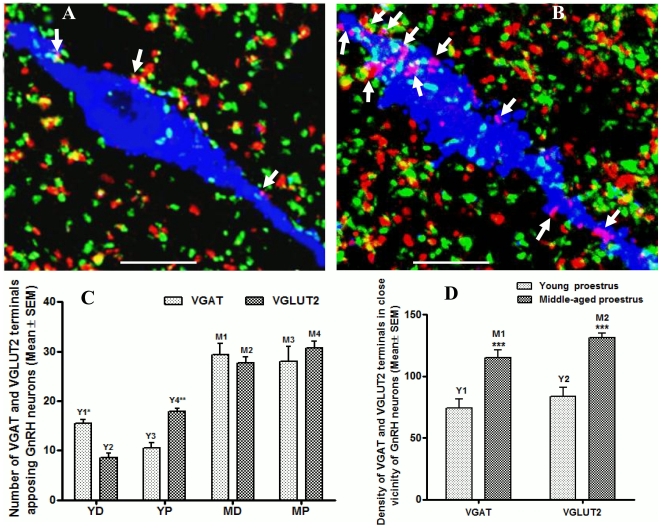
Cycle and age-dependent changes in VGAT and VGLUT2 terminals apposing GnRH neurons. Figure 3A and B, confocal images showing expression of VGAT (red), VGLUT2 (green) and GnRH neuron (blue) from a young (Figure 3A) and middle-aged (Figure 3B) proestrus female rat, respectively. Figure 3C, shows statistical analysis of VGAT and VGLUT2 synaptic terminals apposing GnRH neurons. In young animals at diestrus, average VGAT terminals apposing GnRH neurons were significantly (*P*<0.001) higher (Figure 3C; bar Y1) than VGLUT2 terminals (Figure 3C; bar Y2). However, at proestrus afternoon, VGAT terminals apposing GnRH neurons were significantly (*P*<0.01) decreased (Figure 3C; bar Y3) whereas VGLUT2 terminals (Figure 3C; bar Y4) were significantly increased (*P*<0.001). In middle-aged animals at diestrus, average VGAT terminals (Figure 3C; bar M1) apposing GnRH neurons were not statistically different (*P*>0.05) from the number of VGLUT2 terminals (Figure 3C; bar M2). Similarly, at proestrus, the number of VGAT terminals (Figure 3C; bar M3) apposing GnRH neurons were also not statistically different (*P*>0.05) from the number of VGLUT2 terminals (Figure 3C; bar M4). Furthermore, in either diestrus or proestrus middle-aged animals, average VGAT (Figure3B, and Figure 3C; bars M1 and M3) and VGLUT2 (Figure3B, and Figure 3C; bars M2 and M4) terminals apposing GnRH neurons were significantly higher (*P*<0.0001) compared to young either diestrus or proestrus animals (Figure 3C; bars Y1 andY3 for VGAT and bars Y2 and Y4 for VGLUT2). A further analysis at proestrus showed that in middle-aged animal, density of both VGAT (Figure 3B, and Figure 3D; bar M1) and VGLUT2 (Figure 3B, and Figure 3D; bar M2) terminals in the area in close vicinity of GnRH neurons was significantly (*P*<0.0001) increased compared to young animals (Figure 3A, and Figure 3D; bar Y1 for VGAT and bar Y2 for VGLUT2). Scale in Figure 3 A and B represents 10 µm. Various abbreviations used in Figure 3C are: YD (young diestrus), YP (young proestrus), MD (middle-age diestrus) and MP (middle-age proestrus) animal groups.

Recently it has been shown that the medial subpopulations of GnRH neurons of rPOA are the target of E_2_-induced LH surge in ovariectomized young female rats. In these animals, during the E_2_-induced LH surge, VGAT terminals contacting GnRH neurons were decreased while VGLUT2 terminal contacts were increased [28]. We therefore, examined whether this dual modulation of VGAT and VGLUT2 synaptic terminals apposing GnRH neurons also occurs in naturally cycling young female animals at the time of the preovulatory LH surge on proestrus and whether aging alters this dual modulation in middle-aged animals. We counted VGAT and VGLUT2 positive terminal apposing medial population of GnRH neurons of rPOA from young naïve or breeder rats sacrificed at diestrus (*n* = 6) and proestrus (*n* = 7), and from middle-aged animals sacrificed at diestrus (n = 5) and proestrus (n = 6). Approximately 10–12 GnRH neurons were analyzed per animal and a detailed statistical analysis performed using two-way ANOVA with Bonferroni's posttest analysis is presented in **Figure 3C** **.** The results show that in young cycling animals on the day before LH surge (at diestrus), VGAT terminals apposing GnRH neurons were significantly higher (**Figure 3C**; bar Y1) than VGLUT2 terminals (**Figure 3C**; bar Y2). However, at the time of the preovulatory LH surge (proestrous afternoon), VGAT terminals apposing GnRH neurons were significantly decreased (**Figure 3C**; bar Y3), whereas VGLUT2 terminals were significantly increased ((**Figure 3C**; bar Y4). There was a significant interaction between VGAT and VGLUT2 terminals in young animals across estrous cycle as determined by Bonferroni's posttest analysis. These findings suggest that GABAergic and glutamatergic synaptic contacts on GnRH neurons in cycling young animals are precisely regulated, and that the decrease in GABAergic and increase in glutamatergic synaptic contacts on GnRH neurons on proestrus may serve to facilitate GnRH neuron activation on the day of the preovulatory LH surge.

**Figure 4:**
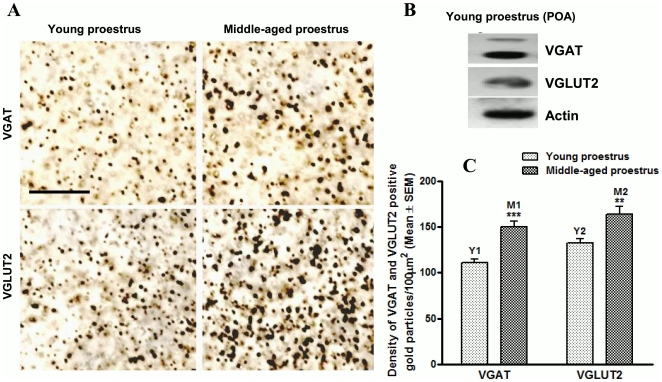
Measurement of VGAT and VGLUT2 terminals in rPOA by immuno-gold labeling. Figure 4A, shows silver-enhanced immuno-gold labeling of VGAT and VGLUT2 synaptic terminals from the rPOA of proestrus young and middle-aged animals. Figure 4B shows western blot detection of VGAT and VGLUT2 proteins from the POA of a young proestrus animal. Figure 4C shows statistical analysis of the density of VGAT and VGLUT2 positive gold particles from proestrous young and middle-aged animals, determined using *Volocity4*. In middle-aged animals, mean density of both VGAT (Figure 4C, bar M1) and VGLUT2 (Figure 4C, bar M2) labeled gold particles was significantly higher (*P*<0.0001) compared to young animals (Figure 4C, bar Y1 for VGAT and bar Y2 for VGLUT2). Scale in Figure 4A represents 5 µm.

It is well known that GnRH neuronal activation and resultant preovulatory LH surge are attenuated on proestrus in middle-aged female rats, which precedes and may facilitate the transition to acyclicity [1], [4], [8], [9], [34], [35]. We thus determined, whether the dual modulation in VGAT and VGLUT2 positive terminals, as observed in young animals, was altered as a function of age in cycling middle-aged rats. The results based on the statistical analysis presented in Figure 3C, shows that the synchronized modulation of VGAT and VGLUT2 terminals apposing GnRH neurons was greatly impaired across estrus cycle stages in middle-aged animals, as there were no significant differences between VGAT and VGLUT2 terminals apposing GnRH neurons at diestrus or proestrous in middle-aged female rats. As shown in **Figure 3C** **,** on diestrus (the day before LH surge) in middle-aged rats the number of VGAT terminals (**Figure 3C**; bar M1) apposing GnRH neurons were not statistically different from the number of VGLUT2 terminals (**Figure 3C**; bar M2). Similarly, at proestrus (at the time of LH surge) the number of VGAT terminals (**Figure 3C**; bar M3) apposing GnRH neurons were also not statistically different (no interaction) from the number of VGLUT2 terminals (**Figure 3C**; bar M4). However, as shown in **Figure 3C** **,** the average density of both VGAT and VGLUT2 terminals apposing GnRH neurons in the middle-aged animals (**Figure 3C**; bars M1-M4) was significantly *increased* compared to young animals (**Figure 3C**; bars Y1-Y4). To further investigate whether the increase in the density of VGAT and VGLUIT2 terminals is restricted to the terminals apposing/contacting GnRH neurons only or is a generalized effect in the area in close vicinity of GnRH neurons, we counted the total number of VGAT and VGLUT2 positive terminals in the close vicinity of GnRH neurons. For this purpose, VGAT or VGLUT2 terminal density was analyzed in all the above *3D* conforcal images using *Volocity4* software. A fixed frame area, as shown in **Figure 3A** and **B** **,** was selected for all the GnRH neurons and either VGAT (red fluorescence) or VGLUT2 (green fluorescence) terminals were counted at a time after hiding the blue channel for GnRH neurons. All the VGAT or VGLUT2 positive terminals present within the frame were included in the analysis, except those touching the boundaries of the frame were excluded. As shown in **Figure 3D**, the mean number of both VGAT and VGLUT2 positive terminals were significantly increased in the area in close vicinity of GnRH neurons in middle-aged proestrus animals (**Figure 3D**; bars M1 and M2) compared to young proestrus animals (**Figure 3D**, bars Y1 and Y2). The increase in average VGAT and VGLUT2 terminal density was further confirmed using silver-enhanced immunogold labeling for VGAT and VGLUT2 in the rPOA on alternate sections. **Figure 4A** shows representative pictures of VGAT and VGLUT2, from the rPOA of proestrous young and middle-aged animals, stained using nanogold-labeled secondary antibodies. **Figure 4B** shows Western blot detection of VGAT and VGLUT proteins from the POA of young proestrous animals. As shown in **Figure 4C**, the average density of both VGAT and VGLUT2 labeled gold particles was markedly elevated in the rPOA of middle-age proestrous rats (**Figure 3C**, bars M1 and M2) as compared to young proestrous rats (**Figure 3C**, bars Y1-Y2). As revealed by one-way ANOVA with Bonferroni's posttest or Student Newman-Keuls posttest, the difference of VGAT and VGLUT2 gold particles density between young and middle-aged animals was highly significant. These results were similar to the results obtained by immunofluorescence staining and confocal microscopy.

## Discussion

This work shows for the first time that VGAT and VGLUT2 contacts on GnRH neurons change during the estrous cycle and with aging in female rats. Specifically, VGAT contacts on GnRH neurons were shown to decrease on the afternoon of proestrus as compared to diestrus in young cycling rats, while VGLUT2 contacts on GnRH neurons increased significantly. One potential interpretation is that the decrease in VGAT-synapses on GnRH neurons on proestrus could reduce inhibition by GABAergic neurons of GnRH neurons, thereby facilitating the GnRH and LH surge. Others workers have reported that levels of the GABA synthesizing enzyme, GAD^67^ is decreased in the rPOA on proestrus in young animals, which has been interpreted to suggest a reduction in GABAergic inhibitory tone occurs on proestrus [18]. Interestingly, administration of the GABA-A receptor antagonist, bicuculline has been shown to advance the timing and amplitude of the LH surge, and to enhance firing of GnRH neurons [19], [36]. In contrast, administration of GABA agonists or use of a Tet-on GAD^67^ viral construct to prevent lowering of GABAergic tone on proestrous afternoon prevented LH surge expression [15], [16]. However, while there is considerable evidence that GABA can exert inhibitory effects on GnRH neuronal firing and secretion, it should be pointed out that there are contradictory studies in the field in which several groups have reported that GABA can actually have *excitatory* effects upon GnRH neurons [20], [21]. Thus, there is some controversy as to the precise role of GABA in the control of GnRH neurons. Obviously, more work is needed in this area. It should be mentioned that previous work showed that VGAT contacts on GnRH neurons are decreased in the rPOA during the estradiol-induced LH surge in young ovariectomized rats [28]. Thus, it is likely that the decrease in VGAT contacts on GnRH neurons observed in our study is due to elevated circulating estradiol levels that occur from diestrus 2 through proestrous afternoon in the young cycling rat, although this remains to be confirmed.

With respect to VGLUT2 contacts, we observed a significant elevation of VGLUT2 contacts on GnRH neurons on proestrus as compared to diestrus in young rats. It is suggested that this elevation of VGLUT2 contacts on GnRH neurons is important for activation of GnRH neurons on proestrus. Indeed, blockade of glutamate neurotransmission on proestrus through use of glutamate receptor antagonists leads to a significant attenuation of the LH surge [13], [14], [37]. Previous work using steroid-treated ovariectomized rats found that the majority of AVPV neurons are dual phenotype neurons (e.g. they are both VGLUT2- and VGAT-positive) and many co-localized estrogen receptor-alpha (ER-α) [28]. The current study extended this observation to the intact rat as we similarly found that the majority of the AVPV neurons in young proestrous rats are dual VGAT- and VGLUT2-positive and colocalize ER-α. However, in the rPOA, we observed that the majority of VGAT and VGLUT2 contacts were juxtaposed, but not superimposed on GnRH neurons, suggesting they are in different terminals. One limitation of our study is that we did not identify the source of the GABAergic and glutamatergic terminals observed to make contact with GnRH neurons, and thus we cannot make any conclusions regarding their origin. Further work is needed to address this issue.

With respect to aging, our study provides evidence that VGAT-and VLUT2 contacts on GnRH neurons are significantly elevated in middle aged proestrous rats as compared to young proestrous rats. Immunological analysis further confirmed the increase in VGLUT2 and VGAT in the rPOA in middle aged proestrous rats. These results suggest that VGAT and VGLUT2 contacts on GnRH neurons increase at middle age. Thus, the pattern for a significant decrease in VGAT contacts on GnRH neurons on proestrus observed in young animals is *lost* at middle age, perhaps leading to unrelieved GABA inhibition of GnRH neurons and thus the attenuated GnRH/LH surge. However, since GABA has also been implicated to have an excitatory role on GnRH neurons, an alternative interpretation is that there may be increased excitatory drive to GnRH neurons in the middle-aged animals. However, administration of the GABA-A receptor antagonist, bicuculline has been shown to partially reinstate the LH surge in middle-aged rats [34], in agreement with an overall inhibitory role of GABA. Nevertheless, one cannot rule out the possibility that the inhibitory influences of GABA may be *indirect* through regulation of other neurons that control GnRH neuron firing. One must also consider potential changes in receptor concentration and receptor type when considering alterations of a neurotransmitter system and its potential impact in aging. Sensitivity of GnRH neurons to a transmitter could change with aging, as has been demonstrated previously for glutamate [4], [38]. We could find no corollary literature on whether GABA sensitivity or GABA receptor expression on GnRH neurons changes in aged animals. A decrease in GABA sensitivity or alteration in GABA receptor levels or type on GnRH neurons with aging could lead to a compensatory *increase* in GABAergic synaptic input to the GnRH neurons, which may explain the increased GABAergic contacts on GnRH neurons we observed in the middle proestrous rat. However, at this point this remains quite speculative, and further studies are needed to address this issue.

With respect to the elevation of VGLUT2 contacts on GnRH neurons that we observed in the middle aged proestrous rats, one could envision this as an attempt to override increased GABA inhibition and/or reflect a potential compensatory mechanism to attempt to counteract a loss of responsiveness of GnRH neurons to glutamate activation. Indeed, we and others have shown that middle-aged rats display significant attenuated GnRH and LH responses to glutamate agonists on proestrus as compared to young animals [4], [38]. Furthermore, a recent study showed that middle-aged proestrous rats have a significant reduction in NMDA1 receptors on GnRH neurons [39], which likely explains the reduced glutamate sensitivity of GnRH neurons in aged rats. Interestingly, recent work by Neal-Perry et. al. [34] showed that there is reduced glutamate and enhanced GABA release in the medial preoptic area in middle-aged rats with attenuated LH surges. Thus, one must additionally consider changes in neurotransmitter synthesis, release and sensitivity, in addition to synaptic contacts on GnRH neurons, to fully understand the mechanisms underlying reduced GnRH neuron activation on proestrus in middle-aged rats.

Finally, it should be mentioned that previous work by Witkin [40] in male rats found an increase in synapses on GnRH neurons in 20 month as compared to young (3 month) old rats, suggesting that increase synaptic contacts to GnRH neurons may also occur with aging in male rats, although the type of synaptic input that was increased was not studied. Our study is the first to our knowledge to address this issue in intact cycling female rats and demonstrates the increase in synaptic appositions in female rats occurs as early as middle-age and involves both VGAT and VGLUT2 synaptic contacts. It is unclear why synaptic appositions to GnRH neurons increases with age. One possibility discussed above is an attempted compensatory mechanism related to decreased sensitivity of GnRH neurons to glutamate and possibly GABA. Another possibility is that astrocyte coverage of GnRH neurons decreases with aging, thereby allowing greater access for synaptic contact. Indeed, Wise et. al. [41] has reported that the surface area of astrocytes in apposition to GnRH neurons is reduced with age. This could lead to dysregulated synaptic control of GnRH neurons and attenuated GnRH release. It could also be that alterations in steroid release, particularly estradiol with aging, could lead to the dysregulation of VGAT and VGLUT contacts on GnRH neurons. Further work is needed to address this issue. In conclusion, the current study provides evidence that VGAT and VGLUT2 contacts on GnRH neurons are altered on proestrus and with aging. These alterations may help explain the induction and control of the preovulatory GnRH and LH surge in young animals, as well as the diminishment of these processes/events observed at middle age in female animals.
